# Nanobiosensing Platforms for Real-Time and Non-Invasive Monitoring of Stem Cell Pluripotency and Differentiation

**DOI:** 10.3390/s18092755

**Published:** 2018-08-21

**Authors:** Intan Rosalina Suhito, Novi Angeline, Sung-Sik Choo, Ho Young Woo, Taejong Paik, Taek Lee, Tae-Hyung Kim

**Affiliations:** 1 School of Integrative Engineering, Chung-Ang University, Seoul 06974, Korea; intanrosalinasuhito@gmail.com (I.R.S.); noa.cms.007@gmail.com (N.A.); sschoo0476@naver.com (S.-S.C.); whymca0@gmail.com (H.Y.W.); paiktae@cau.ac.kr (T.P.); 2 Department of Chemical Engineering, Kwangwoon University, 20 Kwangwoon-Ro, Nowon-Gu, Seoul 0189, Korea; nanotlee@gmail.com; 3 Integrative Research Center for Two-Dimensional Functional Materials, Institute of Interdisciplinary Convergence Research, Chung-Ang University, Seoul 06974, Korea

**Keywords:** stem cells, differentiation, pluripotency, electrochemical detection, Raman spectroscopy

## Abstract

Breakthroughs in the biomedical and regenerative therapy fields have led to the influential ability of stem cells to differentiate into specific types of cells that enable the replacement of injured tissues/organs in the human body. Non-destructive identification of stem cell differentiation is highly necessary to avoid losses of differentiated cells, because most of the techniques generally used as confirmation tools for the successful differentiation of stem cells can result in valuable cells becoming irrecoverable. Regarding this issue, recent studies reported that both Raman spectroscopy and electrochemical sensing possess excellent characteristics for monitoring the behavior of stem cells, including differentiation. In this review, we focus on numerous studies that have investigated the detection of stem cell pluripotency and differentiation in non-invasive and non-destructive manner, mainly by using the Raman and electrochemical methods. Through this review, we present information that could provide scientific or technical motivation to employ or further develop these two techniques for stem cell research and its application.

## 1. Introduction

In recent decades, stem cells have attracted considerable attention in biomedical applications for regenerative medicine and therapy, owing to their powerful ability to differentiate into a wide range of specific cell types in the human body [1,2,3]. Stem cells have been extensively developed for the treatment of diseases, including neural stem cells (NSCs), mesenchymal stem cells (MSCs), and embryonic stem cells (ESCs) [3]. NSCs and MSCs are well known as multipotent stem cells that are able to proliferate in vitro and be differentiated into various types of cells, e.g., neurons, oligodendrocytes, osteoblasts, adipocytes, chondrocytes, etc. [4,5]. Moreover, ESCs are classified as pluripotent stem cells (PSCs) and have the ability to proliferate indefinitely and can generate any specific cell type in the body, such as endothelial cells, skin cells, and many others [6,7,8]. Additionally, it is possible to induce PSCs into multipotent cells, e.g., hematopoietic stem cells (HSCs), MSCs, NSCs, and so on [9]. Thus, with regard to the various lineages of stem cell differentiation, multipotent stem cells are more restrictive in comparison with PSCs. However, the spontaneous teratoma formation caused by any remaining undifferentiated PSCs should be considered as a major issue in terms of its relevancy to regenerative therapies. 

Various existing techniques have generally been used to confirm the successful differentiation of stem cells, such as the chemical staining assay, polymerase chain reaction (PCR), immunocytochemistry analysis, Western blotting, fluorescence-probing, etc. [10,11,12,13]. These common methods are appropriate and effective for the in vitro investigation of stem cell differentiation. Nevertheless, such techniques are also disadvantageous mostly owing to the fixing/lysis step, that is, they are destructive and invasive, which is essential for the staining procedure or for molecular assessment. Concurrently, stem cell differentiation typically requires at least three weeks to generate a specific cell type. Valuable differentiation factors or other molecules to support the generation of specific cells are also required [14], and the differentiated cells undergo certain destructive or invasive techniques to confirm their differentiation. This condition can prevent the implementation of differentiated cells for clinical treatment because the cells are irretrievable after analysis. The development of an analytical technique that enables the precise characterization of stem cell differentiation in a non-destructive manner is desirable, because this could allow the use of stem cells in a more effective and efficient manner.

Raman spectroscopy is a sophisticated tool for the qualitative and quantitative analysis of any molecule within a wide range of applications, and is particularly useful in the field of biology [15,16]. Several previous studies have reported the analysis of stem cell growth and differentiation without using labeling and cell fixation/lysis steps [17,18,19,20]. Subsequently, numerous studies have evaluated the potential of the Raman spectroscopic method in the monitoring of multipotent stem cell (e.g., MSCs and NSCs) differentiation into a specific lineage, such as osteogenesis, adipogenesis, and neurogenesis, even at the single cell level [17,21,22]. Hence, the Raman technique has been highly favored and used in label-free monitoring of stem cell differentiation, and particularly in single cell analysis.

In some cases, PSCs are preferable as an ideal source to obtain many different types of transplantable cells instead of multipotent stem cells, because transplantable cells have the ability to differentiate indefinitely into all cell types. In fact, some concerns have been expressed regarding the presence of undifferentiated PSCs among differentiated cell populations, which could lead to tumorigenesis. Regarding this issue, thorough and rapid monitoring of stem cell pluripotency through stem cell differentiation is necessary prior to conducting safe transplantation. In several studies, preventive efforts have been carried out to avoid the possibility of teratoma formation caused by undifferentiated pluripotent cells, and were particularly based on electrochemical and cell-based sensors [23,24]. Electrochemical sensing could be a strong candidate for non-invasively screening the differentiation of pluripotent stem cells, particularly in real-time and when rapid behavior is exhibited, owing to its capability in detecting electrochemical signals from a very low number of PSCs among the differentiated cells in a non-time-consuming and non-destructive manner.

The development of a highly conductive cell-based chip system has been attracting attention because this platform enables the enhancement of electrochemical signals from the target of interest. Mostly, gold nanoparticles (GNPs) have been used for electrode surface modification in the field of biosensors, owing to their unique characteristics, such as the high electrical conductivity and specific surface area [25,26,27,28]. Interestingly, GNPs have also been employed in the field of Raman spectroscopy, and particularly in surface enhanced Raman spectroscopy (SERS) [29,30]. A conductive platform with a gold nanotopographical structure is reliable in both Raman spectroscopy and electrochemical sensing applications [31]. Thus, its application is highly favorable for monitoring stem cell pluripotency and differentiation owing to its good biocompatibility and non-toxic effects on cell growth, rather than using other materials such as platinum or silver nanoparticles, which could be harmful to the cells under certain circumstances [26].

**Figure 1:**
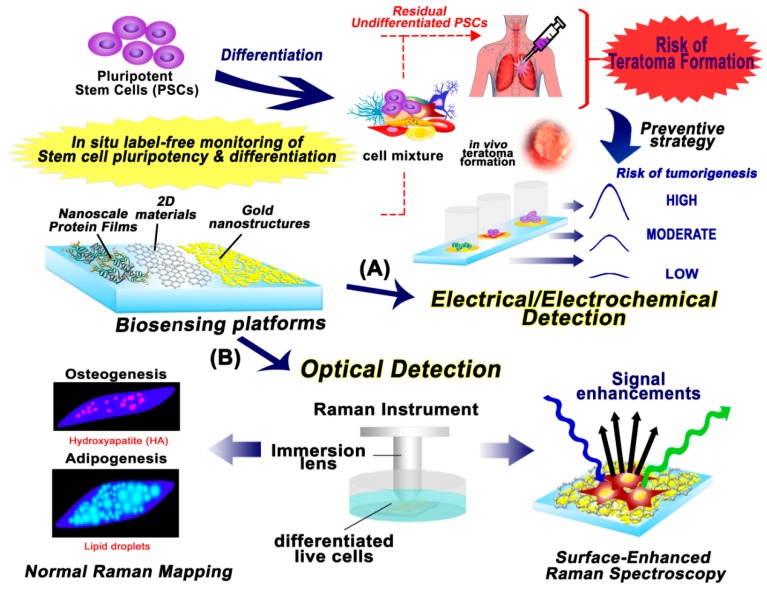
Schematic diagram of nanobiosensing platform for real-time and non-invasive monitoring of stem cell pluripotency and differentiation. This illustration represents the potential of biosensing platforms for: (**A**) electrical/electrochemical detection of stem cell pluripotency and differentiation through electrochemical measurement; and (**B**) optical detection to monitor the successful differentiation of stem cells through Raman spectroscopy.

In this review, we highlight and discuss various studies on the non-destructive and non-invasive monitoring of stem cell pluripotency and differentiation (Figure 1). Specifically, the utilization of Raman spectroscopy and electrochemical sensing techniques, as cell-friendly methods in combination with nanomaterial-functionalized biosensing platforms, were intensively investigated (Table 1). The monitoring of NSC, MSC, and PSC differentiation using both aforementioned techniques is presented and discussed extensively in this paper.

## 2. Monitoring NSC Multipotency and Differentiation

Parkinson’s disease (PD) is a well-known neurological disease that is caused by mass loss of dopaminergic neurons in the special region of the mid brain, substantianigra, and ranks as the second most common disease among all neurodegenerative diseases. Unfortunately, current therapies such as the drug treatment (e.g., L-DOPA, DOPA decarboxylase) and deep brain stimulation are effective only in slowing the progression of PD, not in curing it. To address this critical issue, recent studies on PD have focused on the transplantation of dopaminergic neurons, mostly via converting neural stem cells or pluripotent stem cells (PSCs) into dopaminergic neurons in vitro/in vivo. A number of studies discovered that neural stem cells depend on various factors to induce differentiation in dopaminergic neurons, such as retinoic acid, combinations with the basic fibroblast growth factor (bFGF), leukemia inhibiting factor (LIF), glial conditioned media, B27 treated with sonic hedgehog, fibroblast growth factor 8 (FGF8), and guggulsterone (GS) [32,33,34,35].

To develop a platform that enables label-free and non-destructive monitoring of NSC differentiation, a poly(3,4-ethylenedioxythiophene)-polystyrene(sulfonate) (PEDOT-PPS) modified microelectrode array (MEA) was reported. PEDOT-PSS was chosen because it enhances the electrical signals of target materials via a decrease in the impedance of the electrodes. Both NSC differentiation and migration were found to be successfully monitored, which were shown as a burst pattern and a random noise pattern in the observed graph, respectively. However, this PEDOT-PSS MEA was found to lack the capability to identify the types of cells that result from NSCs [36].

Later, other types of electrical sensing platforms were also reported, including a capacitance array sensor that was developed by Lee et al. [37]. Specifically, changes in the capacitance which occurred on a gold sensing electrode (large counter electrode) indicate the differentiated NSCs. Specifically, the capacitance between a small sensing electrode and a large counter electrode was measured using 100 mV to produce an alternating current (AC) as an electrical source. AC was chosen instead of direct current (DC) owing to its negligible effect on cell functions (e.g., viability, adhesion, and differentiation). NSCs were cultured on the large counter electrode and were induced to differentiate into neuronal and/or glial cells. Interestingly, the authors claimed that slow increases in capacitance with sudden sharp peak formations were observed under the NSC differentiation, as a result of the generation of neuronal and astroglial cells.

**Figure 2:**
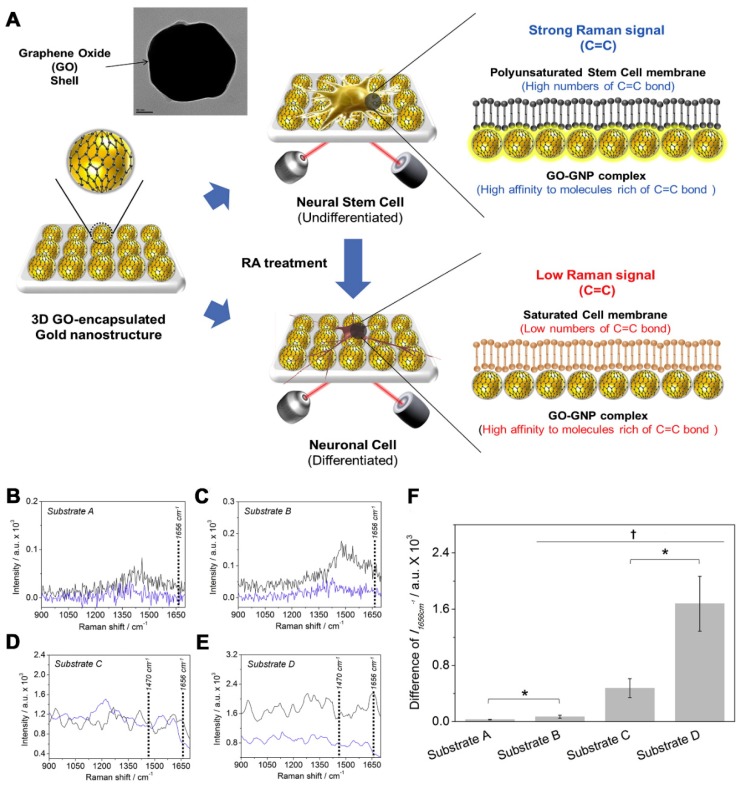
(**A**) Detection strategy to monitor the differentiation of mouse neural stem cells (mNSCs). The cells were allowed to attach to a fabricated substrate; the detection time for the Raman spectra was 1 s in all experiments. Raman spectra of ( **–** ) undifferentiated or ( – ) differentiated mNSCs on: (**B**) Substrate A; (**C**) Substrate B; (**D**) Substrate C; and (**E**) Substrate D. (**F**) Intensity difference of Raman peak at 1656 cm^−1^ (C=C bond) achieved from the undifferentiated mNSCs that were substrated by the differentiated cells († *p* < 0.05, N = 3, ANOVA test and * *p* < 0.05, Student’s *t*-test). (**A**–**F**) Reprinted with permission from [40]. Copyright 2018, Elsevier.

Aside from the electrical tool for the characterizations of NSC differentiation, a Raman-based technique, especially surface-enhanced Raman spectroscopy (SERS), wherein the metallic nanostructures/nanomaterials significantly enhance weak Raman peaks, could be an excellent method as a rapid, non-destructive, and reagent free analysis tool for biological/chemical substances [38]. By using SERS combined with fabricated 3D graphene oxide-(GO)-encapsulated gold nanoparticles, Kim et al. reported that differentiated and undifferentiated NSCs can be identified based on differences in the intensities of the Raman peak (Figure 2A). GO is preferable because of its ability to adhere to the molecules, which contain high C=C bonds, and its outstanding performance in electrochemical analysis [39]. The differences in lipid compositions of the cell membranes, which had a C=C bond peak at 1656 cm^−1^ and a C-H bond peak at 1470 cm^−1^, was higher in the undifferentiated cells compared to the differentiated cells (Figure 2B–F). Undifferentiated cells contained high amounts of C=C bond because of the polyunsaturated cell membrane. Contrary to the differentiated cell membrane, the lipids were saturated; therefore, the Raman signal of the C=C bond was low [40]. Owing to the high sensitivity of SERS, this method is preferable for real-time cell monitoring because it does not disrupt the cells. Additionally, cells can be reused in a subsequent test.

Another substrate for SERS has also been proven as a reliable method for monitoring NSCs in vitro. Gold nanostar has been used as the SERS substrate, and particularly HB1.F3 cells have been used for NSCs. The increasing signal intensities of the peaks in the G/DNA and CO structure in the carbohydrate (690 cm^−1^ and 1120 cm^−1^, respectively), and the decreasing signal intensity of the protein contents and the various DNA contents (730 cm^−1^ and 755 cm^−1^ (Trp), 838 cm^−1^ (Tyr vibrations), 1001 cm^−1^ (Phe), 1310 cm^−1^ (A in DNA), 1540 cm^−1^ (Trp vibrations), 1617 cm^−1^ (C=C Trp and Tyr), and 796 cm^−1^ (PO^2−^ in DNA)) were different in the differentiated and undifferentiated NSCs. From the results, the Raman spectra of the NSCs revealed that the differentiation was within the range of 600 cm^−1^ and up to 1750 cm^−1^. Cyclic voltammetry (CV) has also been used to verify the differentiation of NSCs. In CV detection, the peak of differentiated cells has higher reversibility in comparison with that of undifferentiated cells. This reversibility is related to the nature of membrane hyperpolarization or depolarization, which occurs in the neurons [38]. Based on these results, it has been concluded that the combination of the SERS and CV methods can help to validate the capabilities of SERS with regard to the monitoring of differentiated NSCs with a label-free method.

Another method of enhancing dopaminergic neuron differentiation is the fabrication of pyrolyzed carbon 3D scaffolds (p3D-carbon), which enables the real-time electrochemical detection of dopamine. The 3D environment has been reported to stimulate the rapid and spontaneous differentiation of NSCs into dopaminergic neurons, even in the presence of growth factors. A supporting point for neurites enhances their elongation, and also facilitates the formation of a neuronal network. These are a few of the advantages of p3D-carbon. Moreover, the pillar structure of this substrate is a neurotransmitter trap. Thus, it helps to increase the intensity of the dopamine (DA) signals, which are released by the NSCs. Furthermore, p3D carbon can be used in the direct differentiation and detection of dopaminergic neurons without the need to subculture and add a differentiation factor (DF) [41].

**Figure 3:**
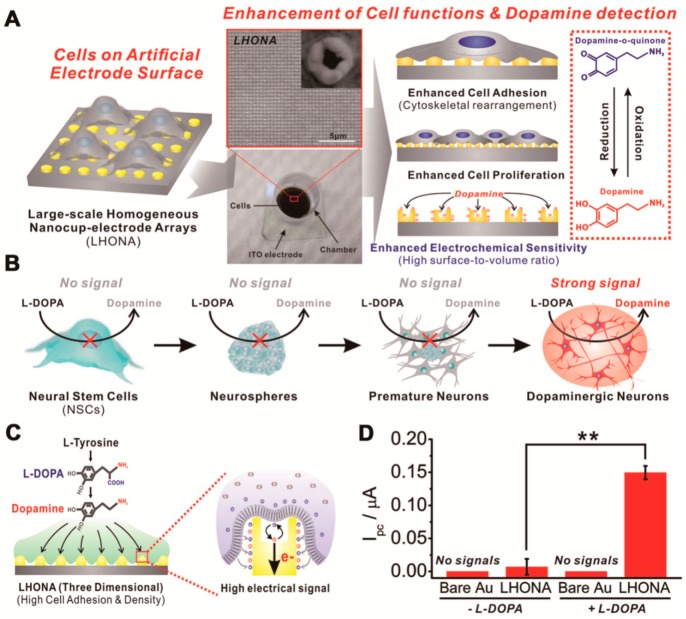
(**A**) Schematic diagram showing the superiority of large-scale homogeneous nanocup electrode arrays (LHONA) as a conductive cell culture platform that enhances major cell functions and electrochemical sensitivity with regard to dopamine detection; both are extremely important for cell-based sensors. The figure shows a cell-based chip used in the detection of dopamine released from the dopaminergic cells composed of the ITO electrode, LHONA, and cell culture chamber. The structure of LHONA as characterized by scanning electron microscopy is also shown in the above image. (**B**) Detection strategy to discriminate the dopaminergic neurons from other types of their progenitor cells by using L-DOPA pretreatment and LHONA as the cell culture platform (working electrode) based on the electrochemical method. Only the cells capable of converting L-DOPA to dopamine can provide distinct redox peaks that can be used as an indicator for the presence of dopaminergic neurons. (**C**) Schematic diagram showing the interaction between the cell membrane and surface of electrodes, which results in the increase of the model dopaminergic cells’ electrical signals in LHONA owing to enhanced cell spreading, adhesion, and proliferation. (**D**) The CV results of PC12 cells on bare Au and LHONA substrates. For LHONA with +L-DOPA and -L-DOPA, the *Ipc* values of dopamine were 0.222 µA and 0.022 µA, respectively. +L-DOPA means that the PC12 cells were treated with L-DOPA prior to the electrochemical analysis. (**A**–**D**) reprinted with permission from [42]. Copyright 2018, Wiley-Blackwell.

Besides the electrical- and/or Raman-based methods, a new technique has been used specifically to detect the dopamine release of NSC-derived dopaminergic neurons using electrochemical detection. Large-scale homogeneous nanocup-electrode arrays (LHONA) are claimed to be a novel cell-based sensing platform that is able to carry out sensitive detection of an electrochemical neurotransmitter from dopaminergic cells and obtain real-time data. LHONA detects the DA’s signal, which is converted by dopaminergic neurons (Figure 3A,B). Other central neural cells, or even neural stem cells, are unable to change L-DOPA to DA. Therefore, with the exception of dopaminergic neurons [42], no other signal exists. By using LHONA, the adhesion of the cells to the surface increases, and produces a better signal than by using a flat surface (Figure 3C,D).

## 3. Monitoring of MSC Differentiation

In the field of biomedical and regenerative therapies, MSCs are frequently used as an agreeable source for therapeutic applications, owing to their multipotency and clinical efficacy (low potential of tumorigenicity) after transplantation [43,44,45,46]. Regarding the differentiation of MSCs into specific lineages (e.g., osteogenesis and adipogenesis), it is worthwhile utilizing analytical techniques, which should be friendly to the valuable differentiated cells that are produced during the differentiation process. Therefore, the yield loss of the differentiated cells could be minimized during patient-specific cell production [17]. With regard to the monitoring function and differentiation of stem cells, common methods, such as PCR, flow cytometry, Western blot, metabolomics analysis, etc. [10,11,12,13,47], are precise and reliable. Paradoxically, these techniques are not appropriate with regard to cell behavior; that is, they are destructive and time-consuming. In line with this evidence, there have been several attempts to detect the fate of MSCs in a non-invasive manner by employing various assessment methods. Such work is useful in the biological investigation of stem cells [17,48,49,50,51,52].

**Figure 4:**
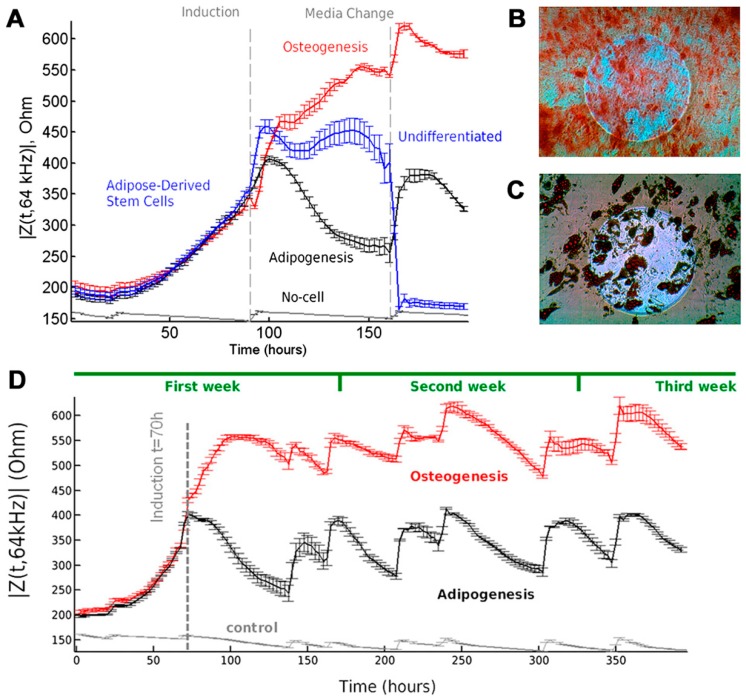
Real-time and label-free monitoring of MSC differentiation into osteoblasts and adipocytes. (**A**) Time-dependent measurement of mean impedance |Z(t,f)|, at 64 kHz for different groups over the course of early induction. MSCs were seeded (t = 0) in multi-well preprinted electrode arrays. At t = 93 h, MSCs were induced toward osteoblasts and adipocytes with an osteogenesis and adipogenesis differentiation medium, respectively. Non-induced MSCs kept growing after confluency until cell detachment occurred. Clear differences in |Z(t,f)| can be observed between all groups. Several days (>14) after induction, histochemical end-point staining was performed to assess if the cells underwent (**B**) osteogenesis (Alizarin red stain) or (**C**) adipogenesis (Oil red O stain). The circular microelectrodes had a diameter of 250 μm and appeared as a bright circle on the micrograph. (**D**) Long-term monitoring of |Z(t,f)| is demonstrated during differentiation over a period of 420 h and plotted at 64 kHz. At t = 70 h, the MSCs were induced toward osteoblasts and adipocytes (n = 3). Reprinted with permission from [52]. Copyright 2018, National Academy of Sciences.

Certain electrochemical-based systems have been reported to detect the behavior of MSCs, including their multipotency and differentiation [50,51,52,53]. Additionally, the electrochemical detection of MSC neurogenesis has been investigated by focusing on the use of a gold nano-dot surface on a chip through cyclic voltammetry (CV) detection of neuronal cells [54]. Moreover, Hildebrandt et al. (2010) proved the advantages of electrochemical impedance spectroscopy (EIS) in the detection of MSC osteogenesis within 2D or 3D cell cultures, because EIS is also one category of electrochemical measurement that is conveniently used in biosensing studies [51,55,56]. Impedance sensing has also been reported as a real-time and label-free approach to oversee the differentiation of MSCs into adipocytes and osteoblasts (Figure 4). In a time-dependent study, apparent impedance for MSC differentiation was characterized as an osteogenic and adipogenic lineage, as shown in Figure 4A. Distinct dielectric property trends have been observed in |Z(t,64 kHz| after a time induction of 93 h for osteogenesis, adipogenesis, and non-induced cells representing the cell responses toward induction treatment. To ensure that the samples are undergoing differentiation, alizarin red S (ARS) and oil red O (ORO) stainings were performed to indicate successful osteogenesis and adipogenesis (Figure 4B,C). Further assessment was conducted for the long-term monitoring of MSC differentiation over a period of 420 h (17.5 days), as shown in Figure 4D. Based on this result, the dielectric properties of the osteo-induced and adipo-induced cells were clearly delineated at multiple frequencies, which indicate the potential of the EIS method in the non-destructive monitoring of MSC differentiation [52].

**Figure 5:**
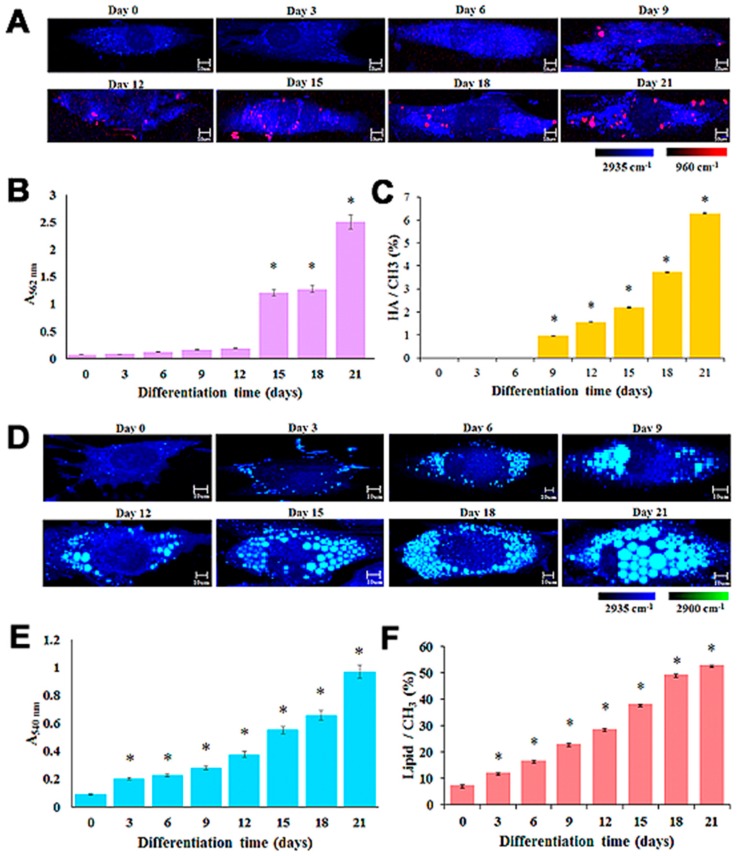
Label-free monitoring of MSC differentiation into osteoblasts and adipocytes in a time-dependent manner: (**A**) Raman mapping of single live osteoblast using the peak at 960 cm^−1^ (referring to the presence of HA); (**B**) ARS quantification staining within an interval time of three days; (**C**) HA/CH_3_ ratio based on Raman mapping analysis; (**D**) Raman mapping of single live adipocyte using the lipid peak at 2,900 cm^−1^; (**E**) ORO quantification staining within an interval time of three days; and (**F**) lipid/CH_3_ ratio based on Raman mapping analysis. Reprinted with permission from [17]. Copyright 2018, Elsevier.

In other aspects, Raman spectroscopy has emerged as an appropriate tool for the assessment of stem cell characteristics and their differentiation into specific cell types [17,21,22,57,58,59,60]. The osteogenesis of MSCs has been successfully monitored by using longitudinal time-lapse Raman imaging throughout the formation of hydroxyapatite (HA) as the osteogenic marker [60]. Based on this evidence, the successful differentiation of other cell types, such as the adipogenic lineage, has been investigated simultaneously with the Raman technique. It has been observed that the sharp Raman spectra of approximately 2900 cm^−1^ indicate the abundance of lipid droplets in adipocytes, and could thus be helpful in generating the Raman mapping of lipid distribution in the cells [58]. Remarkably, an up-to-date study on the label-free monitoring of MSC differentiation has been conducted. The particular focus of this study was on quantitative identification upon the acquisition of Raman mapping in a time-dependent manner of the osteoblast and adipocyte at single cell level for the first time (Figure 5). With regard to osteogenic differentiation, a distinct Raman peak at 960 cm^−1^ indicates the mineralization of HA, and is being considered as a recognition marker for osteoblasts. Figure 5A,D shows the results for the time-dependent Raman mapping of MSC differentiation into osteogenic and adipogenic lineages by using the aforementioned specific Raman spectra (960 cm^−1^ for the osteoblast, and 2900 cm^−1^ for the adipocyte). The quantification of ARS and ORO staining (Figure 5B,E) were performed along with the percentage area of the HA/CH_3_ stretching mode (960 cm^−1^/2935 cm^−1^) and the lipid/CH_3_ stretching mode (2900 cm^−1^/2935 cm^−1^) by referring to the Raman quantification data (Figure 5C,F) to determine the sensitivity of the Raman technique in comparison with that of conventional methods. Based on the data, it was proven that the Raman technique had higher sensitivity to the monitoring of MSC differentiation into the osteogenic lineage, because the HA synthesis could be detected earlier, on the ninth day of differentiation, in comparison with ARS staining, which caused the positive staining of HA two weeks after differentiation. Given the adipogenesis of MSCs, the Raman quantification data corresponded to the ORO staining, where an increase in the amount of lipid droplets was detected on the third day of differentiation [17]. Therefore, the Raman method is more effective and efficient in analyzing the behavior of MSCs considering its non-destructive, label-free, and cell-friendly characteristics, which can overcome the limitations of conventional analyses.

## 4. Monitoring of PSC Pluripotency and Differentiation

Unlike adult stem cells with limited regeneration potency, embryonic stem cells are pluripotent and can differentiate into more than 200 cell types of the adult body [9,61]. Accordingly, embryonic stem cells occupy an enormous part of current stem cell biology and regenerative medicine. However, one of the greatest challenges is to control the differentiation of stem cells, which can be addressed by cell monitoring. In the past, stem cell differentiation has mainly been monitored by applying immunocytochemistry, which is a biological assay. However, this approach does not only require biomarkers or labels, but is also time consuming. Therefore, the need for a technique that could enable the rapid monitoring of stem cell differentiation became obvious [62].

**Figure 6:**
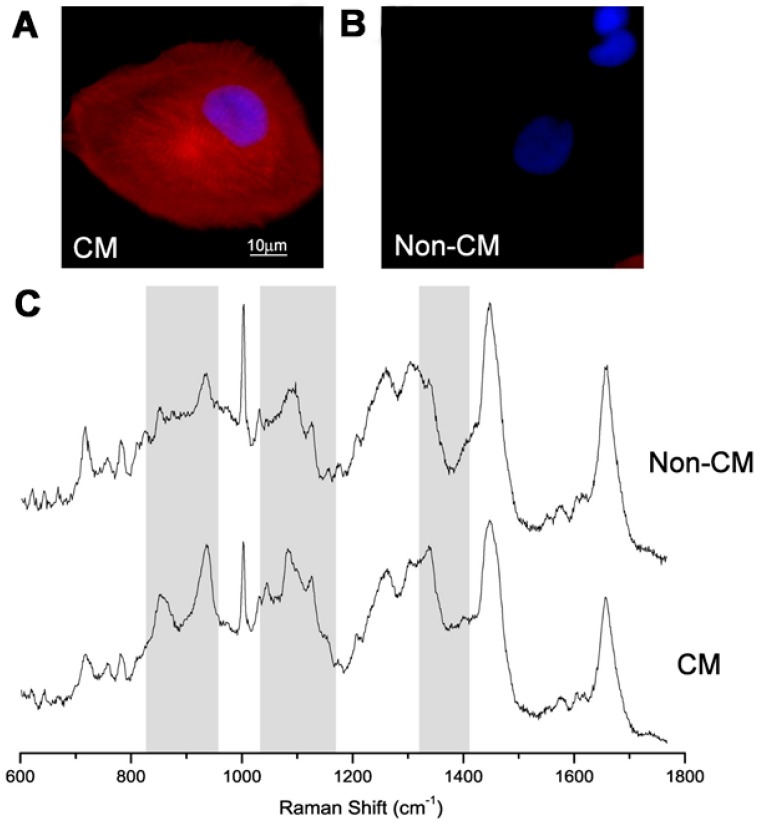
Typical immunostaining images of: (**A**) individual cardiomyocytes (CM); and (**B**) non-cardiomyocytes (non-CM) derived fromhuman embryonic stem cells (hESCs). Cell nuclei DAPI (**blue**); cardiac a-actinin (**red**). (**C**) Raman spectra of the same cells. Reprinted with permission from [67]. Copyright 2018, Elsevier.

Nowadays, techniques such as optical and electrochemical detection are performed to monitor the conditions of living cells [63,64]. In fact, it has been found that optical detection has the advantage of being able to display the changes happening in the cells. However, fluorescence activated cell sorting depends mainly on lineage-specific surface markers that are expressed in the cell membrane. Additionally, there are many cases where these markers are not expressed in the cell surface [65,66]. Because of this problem, fluorescent based detection requires fixation and permeabilization, which makes the cells unusable for treatment purposes. Thus, researchers have started using Raman micro-spectroscopy (RMS), which is an optical detection method that enables the measurement of the molecular characteristics of live cells that have been grown in vitro without requiring labeling or invasive procedures. In this regard, Pascut et al. have used RMS to detect and image molecular markers specific to cardiomyocytes (CMs) derived from human embryonic stem cells (hESCs) in vitro [67]. Because Raman spectroscopy observes vibrational, rotational, and other low-frequency modes in a given system to identify a certain molecule, the authors used RMS to measure the intrinsic chemical differences between different cell types without a labeling process. As shown in Figure 6, they managed to successfully discriminate CMs from other phenotypes with over 97% specificity and 96% sensitivity. Moreover, they concluded that the different levels of glycogen and the lesser contribution of myofibril proteins in CMs are the reasons for the clear discrimination between CMs and other phenotypes. Accordingly, in 2012, the same group further developed a method for measuring molecular changes in intact embryoid bodies (EBs) during invitro cardiogenic differentiation [68]. The group cultured EBs formed by aggregation with a cardiogenic medium in the micro-bioreactors of a Raman microscope and recorded spatially-resolved spectra at 24-h intervals. These spectra exhibited an increase in the intensity of Raman bands, which coincided with the spontaneous beating of EBs recorded by video, seven days after culturing. Thus, it was confirmed that the intensity profile of the Raman bands could potentially be used in the label-free monitoring of EBs in the case of cardiogenic differentiation.

However, it is impossible to miniaturize the optical detection system, and most of the obtained optical signals cannot be transformed into electrical signals and quantified [69,70,71]. To resolve this problem, an electrochemical detection system, which offers both the miniaturization of the entire platform and easy analysis of cell signals, has been employed in stem cell differentiation monitoring. From Matsunaga and Namba’s experiment in analyzing living cells with redox reactions occurring at the interface of living cells in 1984 [71], the electrochemical detection of stem cells has been performed by many scientists to date. Recently, Cheol-Heon Yea et al. reported a newly developed electrochemical cyclic voltammetry (CV) system to determine the differentiation status of embryonic stem cells (ES) in mice [72]. They monitored the differentiation of mouse ES by tracing the electrochemical signal of 1-naphtyl phosphate (1-NP), which is known to dephosphorylate into 1-naphthol, owing to the reaction with one of the embryonic stem cell markers, namely, alkaline phosphatase (AP). Because the electrochemical properties of 1-NP and 1-naphthol are completely different, the researchers were able to clearly distinguish between the ES and the differentiated cells. First, they detected the electrochemical signals of various 1-NP concentrations, and as concentrations increased, the signal was also enhanced. After obtaining this result, they monitored the electrochemical response of undifferentiated ES cells in mice, and as the AP in the undifferentiated mouse stem cells dephosphorylated 1-NP into 1-naphtol, the group treated 1-NP to mouse ES cells for 15 min, and detected the electrochemical signals. Consequently, the stem cell signals decreased as the number of mouse ES cells increased.

**Figure 7:**
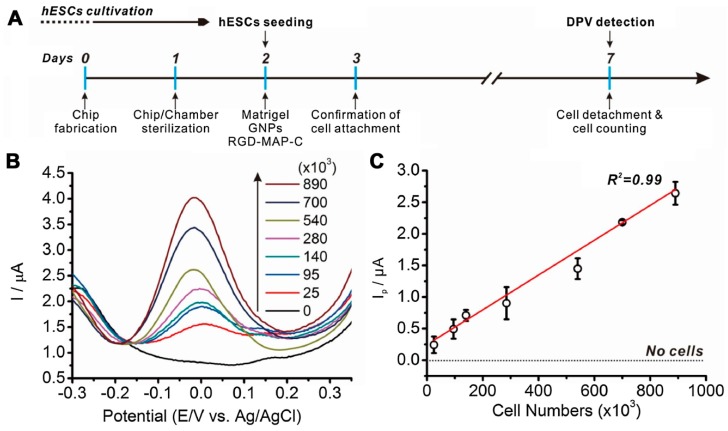
Linear correlations between differential pulse voltammetry (DPV) signals and number of human embryonic stem cells (hESCs): (**A**) brief protocol for cell chip-based electrochemical detection of hESCs; (**B**) DPV signals with indicative number of hESCs, from 25,000 cells to 890,000 cells, presented as an XY graph; and (**C**) linear correlations (R^2^) between number of hESCs and the peak intensity (I_p_) value calculated from the DPV signals. Reprinted with permission from [23]. Copyright 2018, Elsevier.

After a few years, the same group attempted to detect the electrochemical signal of human pluripotent stem cells (hPSCs) to monitor the presence of undifferentiated stem cells in a given sample. Because the risk of teratoma formation from residual undifferentiated pluripotent stem cells (PSCs) is one of the biggest problems in the clinical application of PSC-based therapy, it is important to know how many stem cells exist in an undifferentiated state. To address this, the authors developed a simple electrochemical cell using a gold electrode, and used cyclic voltammetry to detect the electrochemical signal of hPSCs. Thus, they were able to identify a specific electrochemical signal attributable to hPSCs, even under mixed conditions [24]. Based on this study, Ho-Chang Jeong et al. further developed the sensing platform to enhance the sensitivity of the pervious experiment [23]. By exploiting gold nanoparticles (GNPs) and branched arginyl-glycyl-aspartic acid (RGD) peptides to increase the conductibility and adhesion of hESCs, respectively, they fabricated a chip that could detect up to 25,000 human embryonic stem cells, as compared with the previously reported detection of 72,000 cells with clearly linear cell numbers (Figure 7). Accordingly, these groups that performed electrochemical detection for the monitoring of PSCs overcame the drawbacks of optical detection and enabled miniaturization of the detection platform for the electrical quantification of optical signals.

## 5. Conclusions

In this paper, we review numerous studies that have applied non-destructive methods to monitor the differentiation of stem cells (e.g., MSCs, NSCs, and ESCs). Recently, the development of these safe methods has been necessary in stem cell research, owing to the methods’ capability of ensuring undamaged differentiated cells achieved by the non-invasive and non-destructive stem cell differentiation process. Remarkably, Raman spectroscopy and electrochemical sensing have been proven to be effective in the label-free monitoring of stem cell behavior and differentiation. The use of Raman spectroscopy is highly promising, owing to its non-destructive and label-free characteristics with regard to living cells. However, electrochemical methods are also promising candidates as observation tools for stem cell differentiation, because these techniques allow for real-time and rapid detection of the specific cells of interest. Changes in the electrochemical signal can be interpreted as indicators of differentiated cells when compared to the signals of undifferentiated cells. In the field of nanotechnology, substrate fabrication with gold nanoparticles has been found to be highly advantageous with regard to Raman spectroscopy (e.g., SERS) and electrochemical detection because these nanoparticles can enhance signal measurement by increasing the conductivity of the surface area. Therefore, among these findings, the discovery of a biosensing platform for monitoring stem cell pluripotency and differentiation in a non-invasive and non-destructive manner is very interesting. Advanced research on the synergy between Raman spectroscopy, electrochemical biosensing, and nanotechnology could result in the development of a superior platform for stem cell monitoring that could play a very prominent role in biological research, and may thus introduce a new paradigm for stem cell research and its future applications.

## Figures and Tables

**Table 1:** Biosensing Platforms for Real-time and Non-invasive Monitoring of Stem Cell Pluripotency and Differentiation.

Types of Stem Cells	Types of Differentiation	Substrate	Detection Method	Ref.
NSC	Neurogenesis	PEDOT-PSS modified MEA	Electrochemical impedance spectroscopy	[36]
NSC	Neurogenesis	Gold sensing electrode	Capacitance array sensor	[37]
NSC	Neurogenesis	Gold nanostar	Cyclic voltammetry and surface-enhanced Raman spectroscopy	[38]
NSC	Neurogenesis	3D-GO encapsulated gold nanostructure	Raman spectroscopy	[40]
NSC	Neurogenesis	Pyrolyzed carbon 3D scaffolds	Amperometry	[41]
NSC	Neurogenesis	Large-scale homogeneous nanocup-electrode arrays	Cyclic voltammetry	[42]
MSC	Osteogenesis and adipogenesis	Quartz glass	Raman spectroscopy	[17]
MSC	Osteogenesis	Planar electrode-based chip	Electrochemical impedance spectroscopy	[51]
MSC	Adipogenesis and osteogenesis	Gold microelectrode arrays	Electrochemical impedance spectroscopy	[52]
MSC	Neurogenesis	Gold nano-dot surface	Cyclic voltammetry	[54]
MSC	Adipogenesis	Coverslip glass	Raman spectroscopy	[58]
MSC	Osteogenesis	Quartz dish	Raman spectroscopy	[60]
ESC	-	ITO/GNPs/RGD/Matrigel composites	Differential Pulse Voltammetry	[23]
ESC	-	Gold films	Cyclic voltammetry	[24]
ESC	Cardiogenesis	Tissue Culture flask and micro-bioreactors	Raman spectroscopy	[67,68]
ESC	-	Gold electrode	Cyclic voltammetry	[72]
